# Analysis of Methylation‐driven Genes in Pancreatic Ductal Adenocarcinoma for Predicting Prognosis

**DOI:** 10.7150/jca.53208

**Published:** 2021-09-09

**Authors:** Zihan Zhang, Rui Zhu, Wentian Sun, Jun Wang, Jin Liu

**Affiliations:** 1 Lab for Aging Research, National Clinical Research Center for Geriatrics, State Key Laboratory of Biotherapy, West China Hospital, Sichuan University, Chengdu, Sichuan, China.; 2 State Key Laboratory of Oral Diseases, National Clinical Research Center for Oral Diseases, Department of Orthodontics, West China Hospital of Stomatology, Sichuan University, Chengdu, Sichuan, China.; 3 State Key Laboratory of Oral Diseases, National Clinical Research Center for Oral Diseases, Department of Prosthodontics, West China Hospital of Stomatology, Sichuan University, Chengdu, China.

**Keywords:** Pancreatic ductal adenocarcinoma, DNA methylation, Proportional hazards models, Survival analysis, Prognosis

## Abstract

**Purpose:** Considerable variations in methylation profile have been found in various cancers to modulate tumorigenesis and affect prognosis. To provide a theoretical basis for early detection, prognosis evaluation and targeted treatment for patients with pancreatic ductal adenocarcinoma: PDAC, this study identified methylation-driven genes in PDAC and explored their prognostic performance.

**Methods:** The methylation, expression and clinical data of PDAC patients were extracted from TCGA database. Based on the β-mixture model of the MethylMix R package, the differential methylation status and connection between methylation and expression degree were examined to screen out methylation-driven genes in PDAC. COX analyses and lasso regressions were applied to construct a linear risk model based on methylation-driven genes. Univariate and multivariate analyses were performed to ensure the risk model was an independent prognostic factor. Joint survival analyses of methylation and gene expression were conducted to explore the prognostic value of component genes. The methylation sites in the key genes were also investigated.

**Results:** A total of 118 methylation-driven genes in PDAC were identified, and two genes (FOXI2, MYEOV) constituted the risk model whose AUC was 0.722 at one year of overall survival rate, displaying a better performance on survival prediction than other clinical features. Further survival analyses demonstrated that the expression of MYEOV and combined methylation and expression levels of the genes MYEOV and FOXI2 can be potential biomarkers for survival prediction and targets of drug manipulation of PDAC patients. Close relationships were discovered between two sites in MYEOV and one site in FOXI2 and the prognosis of PDAC patients.

**Conclusion:** Concentrating on DNA methylation, our study identified potential biomarkers and developed a reliable short-term predictive model for prognosis of PDAC patients.

## Introduction

Pancreatic cancer has been one of the most lethal malignancies with an overall 5-year survival rate less than 9%, which is the lowest amongst all cancer sites 1. Though the incidence of pancreatic cancer is relatively low, only ranked as the 14^th^ most common cancer, its high fatality rate still poses a great threat to people's life. Due to initially insidious nature and lack of specific symptoms, pancreatic cancer is often diagnosed at an advanced stage, and only 20% of patients can still benefit from surgical resection^2^. Despite decades of considerable researches, no significant improvement has been made in survival rate, accompanied by a still rising incidence 3. Pancreatic ductal adenocarcinoma: PDAC is the most common type of pancreatic cancer, thus it is of great importance from the social perspective to screen out PDAC and achieve early diagnosis 4, for the early initiation of treatment always guarantees a less invasive approach and better prognosis. The development of new biomarkers for PDAC may lead to early diagnosis and thereby potential for more successful treatments 5. Another problem confronting physicians is the lack of predictive tests for prognosis, so treatment protocols can only be made based on the basic condition of patients and the disease staging 6. Therefore, to develop personalized targeted treatment, there is an urgent need to construct a reliable and practicable method for survival rate assessment.

Over the past few decades, there has been a sustained research activity in the cancerous genomic alterations, and it has become increasingly apparent that epigenetics plays a critical role in tumorigenesis. As a core element of epigenetics, DNA methylation has an impact on multiple cellular processes including cell differentiation, genome stability, and gene imprinting 7, 8. Aberrant DNA methylation can be divided into two types: hypermethylation and hypomethylation. DNA hypermethylation means the accumulation of methylation that mainly causes transcriptional suppression and reduced gene expression, while DNA hypomethylation represents less DNA methylation that often leads to disorder of chromosome stability or increased aneuploidy 9. The emergence of aberrant DNA methylation is frequently detected in the promoter regions of transcription factors, resulting in the initiation and proliferation of cancers^10^. Given the strong association between methylation and tumorigenesis, it is vital to better understand the DNA methylation pattern to identify corresponding biomarkers for early diagnosis, prognosis and therapy. At the early stage of cancerization, epigenetic alterations can take place even before the occurrence of genetic changes, thereby becoming potential biomarkers for early diagnosis and prevention of cancers 11. While genetic alterations are irreversible, epigenetic changes are reversible 12, making pharmacological manipulations of methylation process and specific pathways promising in the future 13.

As a crucial instrument for researches on molecular mechanisms, bioinformatic analysis has been broadly utilized to explore the pathogenesis of tumors on a molecular level and to identify potential biomarkers for early diagnosis, treatment and prognosis 14. It is of great importance to identify novel driver genes associated with PDAC through bioinformatics analysis for the establishment of prognosis evaluation protocol and targeted pharmaceutical treatment.

For now, there have been studies about methylation-driven genes for a few types of cancer 14, 15, but no previous study has focused on the expression profiles of differentially methylated genes to construct a Cox model for predicting survival rate of PDAC patients. In this study, expression and methylation profiles of PDAC were downloaded from The Cancer Genome Atlas (TCGA). TCGA is a worldwide open database containing genomic and clinical data of various cancers, serving as a convenient and practical tool in the study of tumorigenesis 16. The exploration of data was performed using the MethylMix algorithm, an R package aiming to find disease-specific hypomethylated and hypermethylated genes based on a β-mixed model 17. After the identification of methylation-driven genes, the Cox model, lasso regression and Kaplan-Meier survival analysis were used to construct risk model and examine the prognostic value of the model and methylation of relevant genes and sites, providing a solid foundation for future research about early detection and personalized medicine.

## Methods

### Data processing and identification of methylation-driven genes

Gene methylation and expression data for PDAC in this study were obtained from the TCGA platform. The inclusion criteria for patients in the TCGA database are as followed: Primary, untreated tumor; Frozen, sufficiently sized, resection samples; samples composed of at least 80% tumor nuclei. All available patients' data in the TCGA database was included in the study. DNA methylation data, generated by the Illumina Infinium Human Methylation 450 bead chip, consisted of 149 pancreatic tumor samples and eight adjacent non-tumoral pancreas samples. The methylation degrees were measured by preprocessed β value, a parameter defined as the ratio of methylated to unmethylated alleles intensity ranging from zero (completely unmethylated) to one (completely methylated). Gene expression data, acquired through RNA sequencing (RNA‐seq), contained 142 tumor samples and two non-tumoral samples. All data were normalized using the limma package in R software (version 3.6.1). Subsequently, clinical data of PDAC patients were also extracted for further survival analyses.

The MethylMix package requires tumor samples with both gene expression and methylation data for combined transcription and methylation analysis, thus unqualified tumor samples were excluded. Subsequently, gene expression and methylation data of tumor samples as well as methylation data of non-tumoral samples were analyzed through the MethylMix algorithm to identify methylation-driven genes in PDAC. Methylation-driven genes should meet two requirements: (1) differentially methylated between non-tumoral and tumor samples, (2) negative correlations between gene expression and methylation status. First, based on the β-mixture model, the Wilcoxon rank sum test was employed for the identification of disease-specific hypomethylated and hypermethylated genes. Statistical analyses were performed applying a significance level of FDR=0.05. Next, the linear regression model was utilized to examine the correlations between gene methylation and expression levels. Genes with the correlation coefficient lower than -0.3 were included. A heatmap was generated by the pheatmap package to show the bidirectional hierarchical clustering results of all methylation-driven genes.

### Functional and pathway enrichment analyses of methylation-driven genes

The obtained methylation-driven genes were submitted to The Database for Annotation, Visualization and Integrated Discovery (DAVID) for Gene Ontology (GO) term analysis and ConsensusPathDB website for functional and pathway enrichment analyses. DAVID (http://david.abcc.ncifcrf.gov/), an open online platform, serves as a functional interpretation tool of large gene lists to further explore their biological mechanisms and associations between molecules 18, 19. ConsensusPathDB (http://cpdb.molgen.mpg.de/), a database containing a comprehensive integrated interaction network, biochemical pathways and functional data in Homo sapiens, provides researchers with a deeper understanding of cellular processes in diseases 20, 21.

### Predictive risk model construction and risk score calculation

The following procedures were conducted to select methylation‐driven genes to construct the model. First, univariate Cox analysis was performed on all 142 cancer samples with survival package to screen out the genes with prognostic implications in PC. The cut-off point was P<0.05. Next, the least absolute shrinkage and selection operator (LASSO) method was applied with the glmnet R package to select the most useful genes weighted by their coefficients respectively. LASSO is a linear regression technique that combines regularization and variable selection, which can increase the precision of the regression model by preventing overfitting. Finally, the remaining genes with non-zero coefficient were included in subsequent multivariate Cox analysis to develop the prognostic model with survival package.

After the Cox model was constructed, all samples were evaluated by this model. The prognostic risk index score was defined as follows:


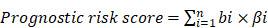


In this formula, β represented the expression level of genes in the model, and b stood for their coefficients in multivariate analysis. The prognostic risk score was calculated for all samples as described in the equation.

### Assessment of the model and component genes

Using the median of the prognostic risk value as a cut-off point, patients were split into two groups: high-risk and low-risk groups. The Kaplan-Meier survival curves of patients in high and low risk groups were plotted to present the overall survival rate using survival package. Log-rank tests were used to assess the significance of the difference in the overall survival rate between the two groups. Time-dependent ROC curves were used to confirm the reliability of the prognostic model. The gene expression data and the corresponding clinical data of GEO datasets GSE62452 and GSE28735 were downloaded to test the reliability of the Cox model. The expression data of two datasets were merged and batch normalized using the sva and limma R packages. After the prognostic risk values were calculated for all patients with PDAC, patients were split into high-risk and low-risk groups according to the median risk score. The Kaplan-Meier survival analysis and time-dependent ROC analysis were performed on the GEO datasets as well.

To test whether the risk score was an independent prognostic factor of PDAC, we integrated the risk score and clinical data of patients in from the TCGA database containing age, sex, grade, stage and TMN classification. Univariate and multivariate stratified analyses were performed with the survival package to ensure the reliability and feasibility of the predictive model. Forest plots were generated with the forestplot package to show the hazard ratio (HR) and P value of each factor.

The prognostic performance of every gene in the model was assessed by both survival analysis on methylation levels and joint survival analyses on combined gene expression and methylation status, in which genes significantly related to patient survival were identified as key genes. To further investigate internal prognosis-related methylated sites of these key genes, the correlations between methylation of sites and gene expression were assessed using the linear model. Sites with a coefficient less than -0.3 and P<0.05 were considered to have an impact on gene expression. Next, The Kaplan‐Meier curves were generated for pertinent methylation sites of keys genes by the survival package to evaluate their prognostic value. All analyses were judged statistically significant at P=0.05.

## Results

### TCGA data analysis and acquisition of methylation‑driven genes in PDAC

**Figure 1:**
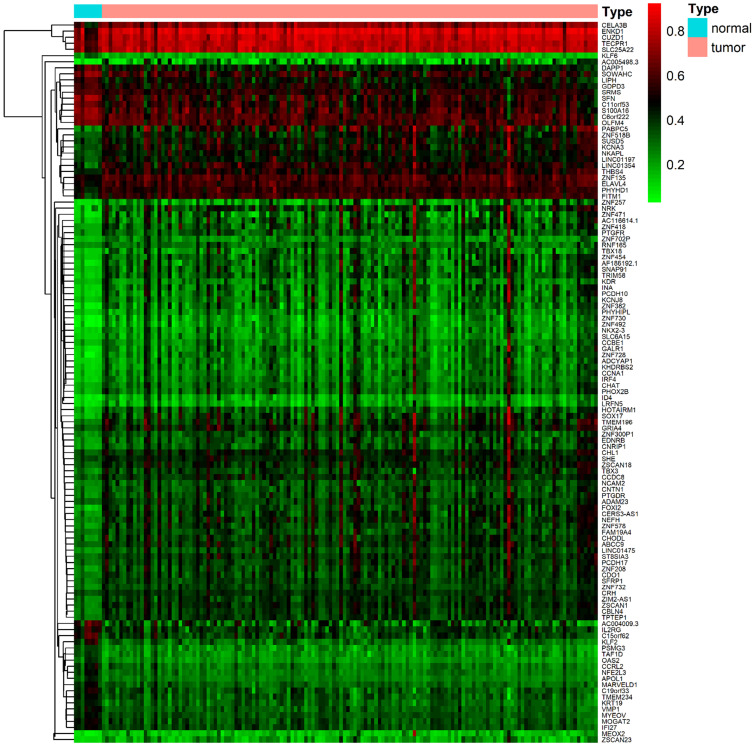
The heat map of methylation-driven genes in PDAC. The colors from red to green presented a tendency from hypermethylation to hypomethylation. The rectangular bar on the top showed the sample types, with blue representing adjacent non-tumoral pancreas tissues and red for PDAC samples.

DNA methylation data were collected from 157 samples, including 149 cancer samples and ehight non-tumoral samples. Gene expression data were obtained from 144 samples, including 142 tumor samples and two non-tumoral samples. Acquired data of PDAC from the TCGA database were normalized by the LIMMA package. Then, the MethylMix package was utilized to perform correlation analysis, β mixed model development and Wilcoxon rank test. The cut-off criteria for methylation-driven genes was set as |logFC| > 0, FDR < 0.05, Cor < -0.3. A total of 118 methylation-driven genes were identified, including 29 hypomethylated genes and 89 hypermethylated genes (Additional file 1, table S1). The result of the bilateral hierarchical cluster analysis was illustrated in the heatmap (Figure 1).

### Functional enrichment and pathway analyses of methylation‑driven genes in PDAC

**Figure 2:**
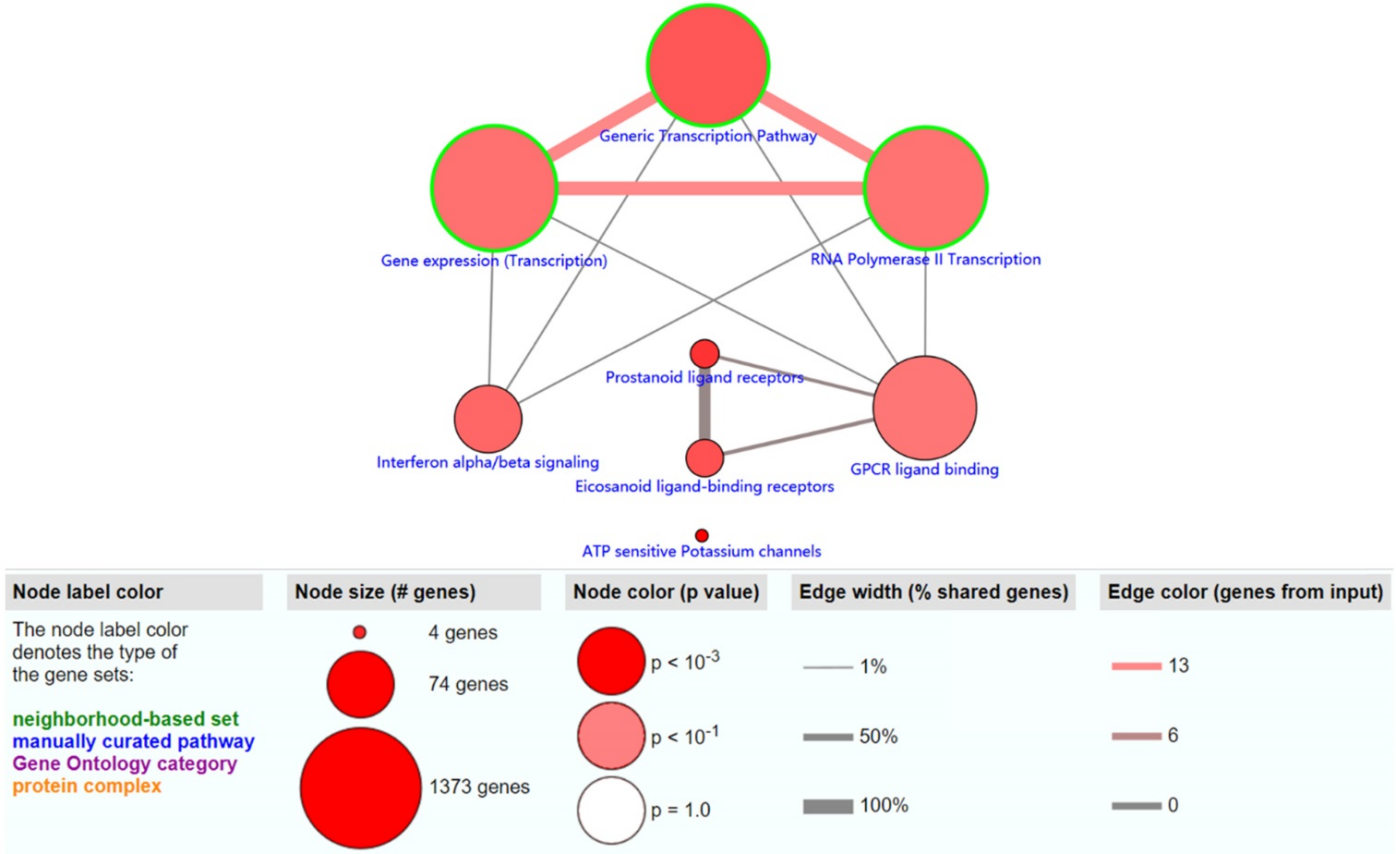
The significantly enriched pathways (P < 0.01) of methylation-driven genes.

The objective of this section was to investigate the molecular mechanism of methylation-driven genes in PDAC. GO functional enrichment and pathway analyses of these genes were performed with DAVID and ConsensusPathDB online platform. GO terms with FDR<0.1 and pathways with P<0.01 were included. GO functional enrichment analysis results showed that the biological processes (BP) of these genes were mainly involved in transcription, DNA-templates; molecular functions (MF) included transcription factor activity, sequence-specific DNA binding and RNA polymerase II core promoter proximal region sequence-specific DNA binding, while the result of cellular component (CC) was not statistically significant (Table 1). Pathway analysis revealed that the methylation-driven genes were mainly enriched in the gene expression (transcription), RNA polymerase II transcription, and generic transcription pathways (Figure 2).

### Development of the risk model based on methylation‑driven genes in PDAC

**Figure 3:**
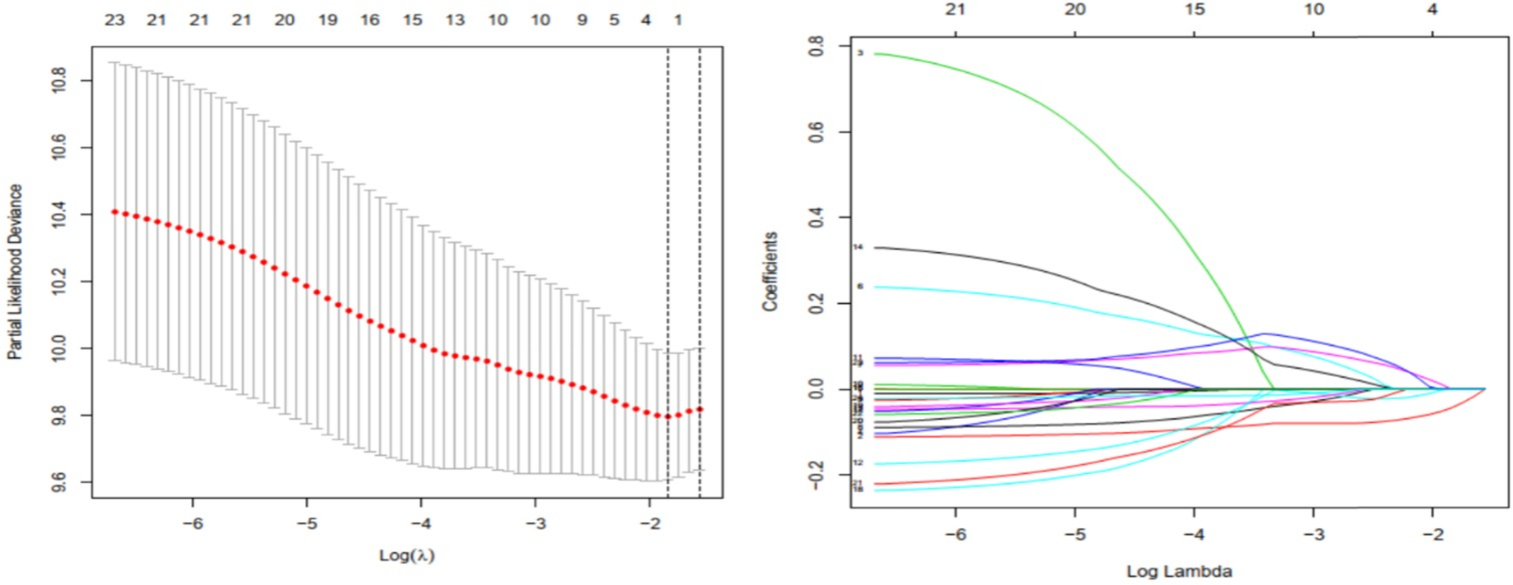
Gene selection through LASSO logistic regression model. (a) The tuning parameter (λ) was determined using 1000-fold cross-validation with minimum standard. Optimal values according to the minimum criteria are denoted by dashed vertical lines. (b) LASSO coefficient profiles of 24 genes were produced against the log (λ).

Univariate regression analysis was performed on acquired methylation-driven genes to identify genes associated with prognosis in PDAC. Table 2 shows the identification of 24 methylation‐driven genes that were related to prognostic risk in PDAC. Lasso regression analysis was used to screen out genes that were either insignificant or redundant for multivariate Cox analysis. Two genes (FOXI2, MYEOV) resulted in non-zero coefficients after fitting into the LASSO regression model (Figure 3). FOXI2 and MYEOV remained to construct an assessment model to predict prognosis of PDAC after multi-factor analysis, and the prognostic risk score= (-0.12878* expression level of FOXI2) + (0.161623 * expression level of MYEOV). The basic characteristics of FOXI2 and MYEOV as methylation-driven genes were shown in Additional file 2: Figure S1 and Additional file 3: Figure S2.

### Prognostic values of the model and the three genes constructing the model

**Figure 4:**
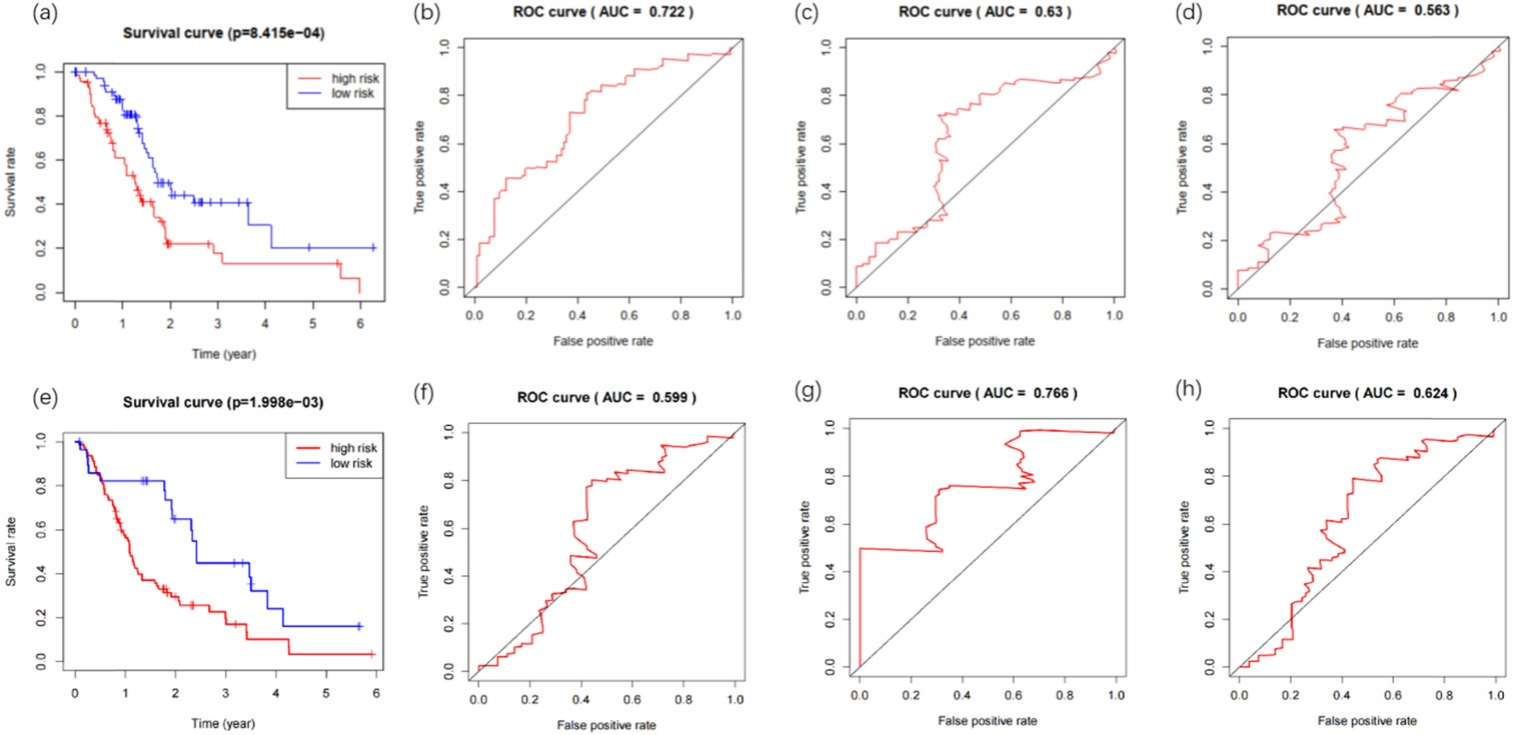
Kaplan-Meier and ROC curves for the constructed prognostic risk model. (a) The difference between high and low risk patients from the TCGA database was measured by the log-rank test. (b) Time-dependent ROC curve for the 1-year overall prognosis prediction of patients from the TCGA database. (c) Time-dependent ROC curve for the 3-year overall prognosis prediction of patients from the TCGA database. (d) Time-dependent ROC curve for the 5-year overall prognosis prediction of patients from the TCGA database. (e) The difference between high and low risk patients from the GEO database was measured by the log-rank test. (f) Time-dependent ROC curve for the 1-year overall prognosis prediction of patients from the GEO database. (g) Time-dependent ROC curve for the 3-year overall prognosis prediction of patients from the GEO database. (h) Time-dependent ROC curve for the 5-year overall prognosis prediction of patients from the GEO database.

The clinical data for 142 PDAC patients were downloaded from the TCGA database, including survival data (survival time and survival status), age, gender, grade, stage, TNM classification and so forth (Additional file 4). The prognostic risk values of patients containing survival data (n=142) were calculated, and patients were divided into low-risk (n= 71) and high-risk (n= 71) groups according to the median value. Kaplan-Meier survival curves analysis of the two groups demonstrated a statistically significant overall survival rate discrepancy (Figure 4a). The time-dependent ROC curve was plotted to assess the prognostic performance of the predictive risk model. The area under curve (AUC) of the Cox model constructed by methylation-driven genes was 0.722, 0.63, 0.563 respectively at one (Figure 4b), three (Figure 4c) and five (Figure 4d) years of overall survival rate, indicating that this method can deliver reliable short-term assessment of PDAC prognosis. The extremely low five-year survival rate of PDAC may account for the reduced reliability as time grows. The Cox model constructed in our study was further tested in two GEO datasets (GSE28735 and GSE62452). The gene expression and clinical data (Additional file 5) of 45 patients were included in GSE28735 and 69 patients were included in GSE62452. After the merger of these two datasets and deletion of patients with incomplete survival information, the data of 108 patients with PDAC (42 patients from GSE28735 and 66 patients from GSE62452) was obtained to perform the analysis. Results proved that the method also perform with sufficient reliability when used in other datasets, as the Kaplan-Meier survival analysis of the high-risk and low-risk groups demonstrated their difference in the overall survival rate was statistically significant (Figure 4e), and the AUC of the Cox model was 0.599, 0.766 and 0.624 respectively at one (Figure 4f), three (Figure 4g) and five (Figure 4h) years of overall survival rate.

**Figure 5:**
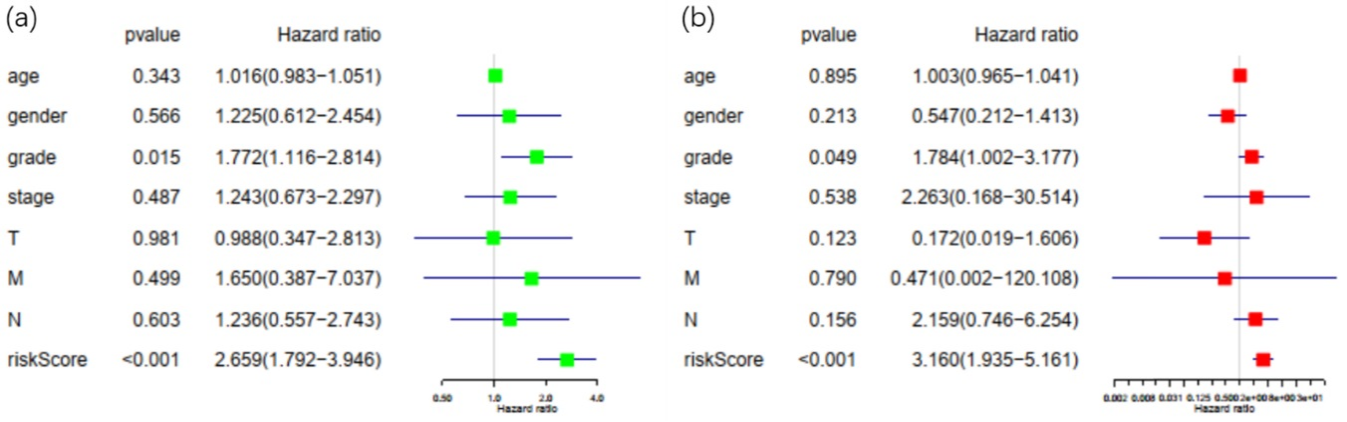
Univariate and multivariate analysis of possible prognostic elements in PDAC. (a) Forest plot of univariate survival analysis. (b) Forest plot of multivariate survival analysis. Abbreviation: HR: hazard ratio. T: description of primary tumor site. N: description of regional lymph node involvement. M: description of the presence or otherwise distance of metastatic spread.

Patients in the TCGA database with incomplete or unknown clinical data were excluded, and a total of 67 patients were eligible for later clinical analysis. Risk score calculated by the Cox prognostic model along with all the clinical factors mentioned above was then imported into R software for univariate and multivariate analysis with survival package (Figure 5). With P<0.05 as the cut-off point in multivariate analysis, only tumor grade and risk score displayed sufficient reliability for prognostic prediction. These results demonstrated that the risk score was a statistically significant independent factor for prognostic evaluation, with the highest hazard ratio among all factors. Solely based on clinical features, existing methods for prognosis evaluation proved to be inefficient, while the risk score had better performance over grade, stage and TNM classification of PDAC.

**Figure 6:**
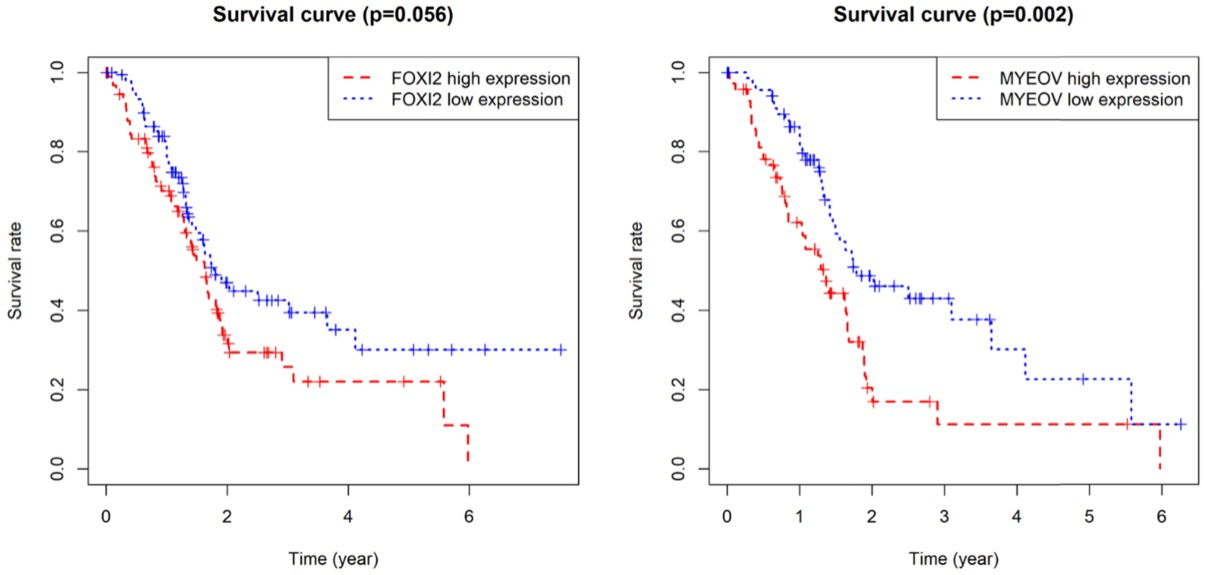
Kaplan-Meier survival curves for the key genes. (a) Survival analysis based on the expression level of FOXI2. (b) Survival analysis based on the expression level of MYEOV.

**Figure 7:**
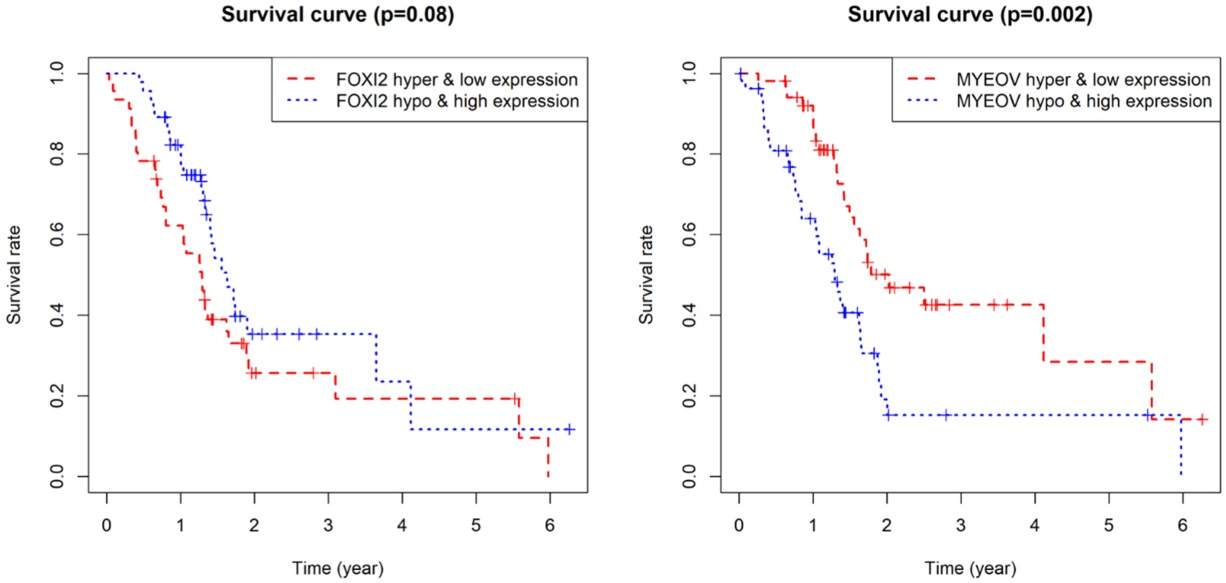
Joint survival analysis for the key genes. (a) Joint survival analysis based on the methylation and expression degree of FOXI2. (b) Joint survival analysis based on the methylation and expression degree of MYEOV.

As for the two component genes in the model, survival analyses showed that while the expression of FOXI2 alone is not of prognostic value, the aberrant expression of MYEOV have significant impacts on prognosis (Figure 6). Joint survival analyses explored the influence of combined methylation and expression on prognosis, demonstrating hypomethylation and high expression of FOXI2 were associated with favorable prognosis, while hypomethylation and high expression of MYEOV were indicative of poor survival rate (Figure 7).

**Figure 8:**
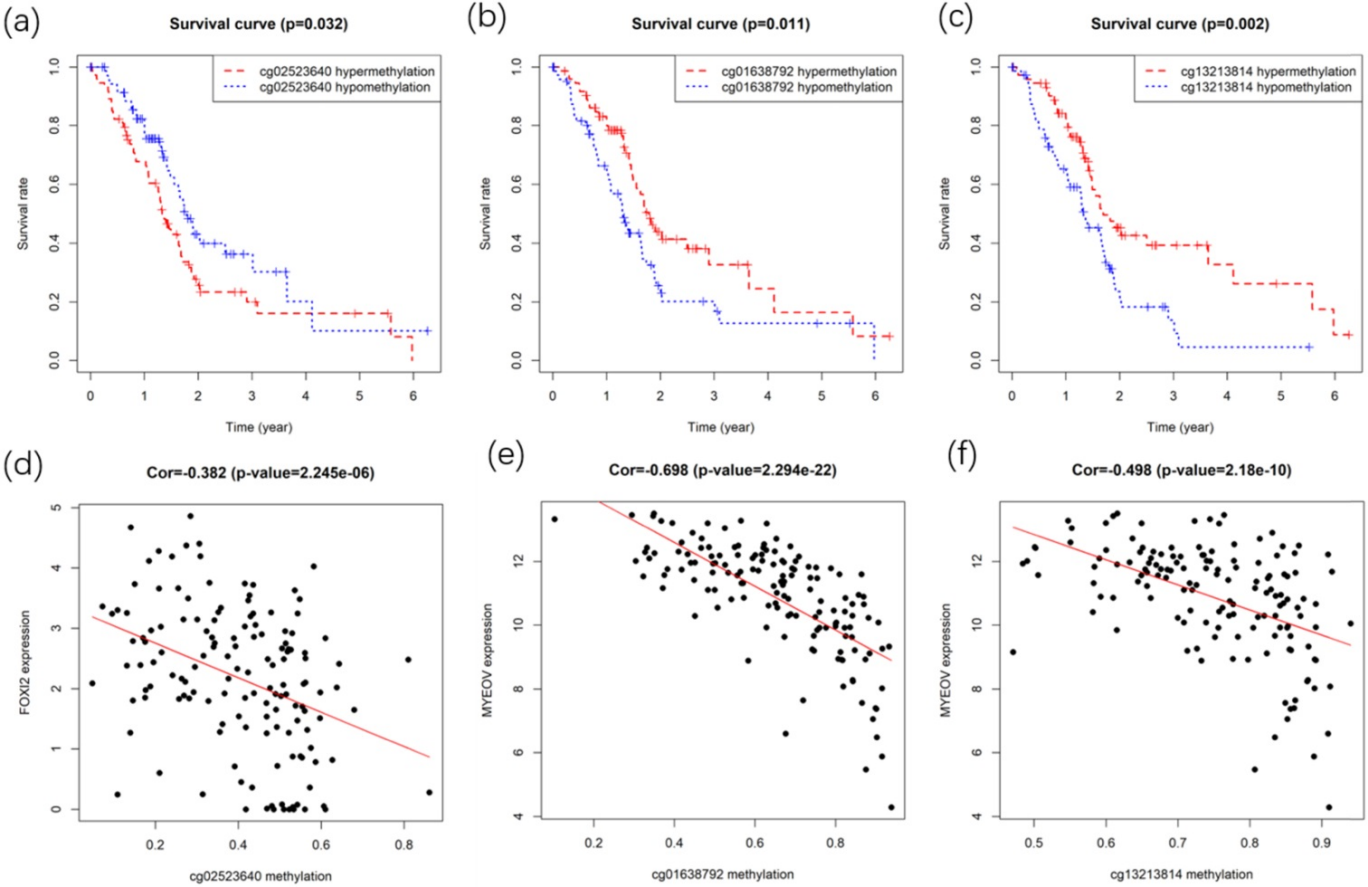
Survival and correlation analysis for the methylation sites in key genes. (a) Kaplan-Meier survival curve of methylation site in the FOXI2. (b,c) Kaplan-Meier survival curves of methylation sites in MYEOV. (d) Correlation between site methylation and FOXI2 expression. (e,f) Correlation between sites methylation and MYEOV expression.

After identifying gene FOXI2 and MYEOV as key genes with prognostic values, their methylation sites were analyzed. Gene expressions of FOXI2 and MYEOV were found to be negatively correlated with the methylation degree of various sites. Kaplan-Meier survival analyses of these sites were performed to identify methylation sites associated with the overall survival rate of PC. Finally, one site of gene FOXI2 and two sites in gene MYEOV (Figure 8) were found to have impacts on both the expression of genes and the prognosis of PDAC.

## Discussion

Pancreatic cancer is a highly malignant tumor with a high mortality rate that almost catches up with the disease incidence 22. To provide new insights into the improvement of PDAC patients' quality of life and prognosis, the molecular pathogenesis of PDAC needs to be studied in-depth for the identification of specific driving genes as early detector and prognostic markers. In recent years, there has been an increased recognition that more attention needs to be paid to epigenetic alterations of tumor cells, which is another major source of tumor cells evolution and resistance to chemotherapy and immune surveillance 23. Gene methylation is a crucial signaling tool regulating normal genomic function, but some abnormal methylation events can act as driving factors in mediating carcinogenesis 24. As the first epigenetic change identified in neoplasia, aberrant DNA methylation usually contributes to the progress of several diseases by the disturbances of signal pathways 25.

A body of study has shown that alterations of DNA methylation can provide valuable new insights into early diagnosis, prognosis assessment and clinical applications for pancreatic cancer. Nishizawa N et al. carried out three experiments to confirm that promoter DNA methylation of CDO1 was specific for PDAC 26. Li XB et al. verified that DNA methylation of BNC1 and SEPT9 gene in plasma cell-free DNA could be utilized to develop a non-invasive detection method for pancreatic cancer 27. Promoter methylation of ADAMTS1 and BNC1 in blood was identified by Eissa MAL et al. as potential biomarkers for early detection of pancreatic cancer 28. Curia MC et al. found out high-status promoter methylation of PCDH10 could be meaningful to identify PDAC patients with high risk of disease deterioration 29. Three hypomethylated genes (SULT1E1, IGF2BP3, MAP4K4) were found by Huiming C et al. to be associated with poor overall survival in pancreatic cancer patients 30. Therefore, it is expected that advancements in the early diagnosis, personalized therapy and risk evaluation for PDAC will be made through the understanding and modification of DNA methylation profile.

The main objective aim of this work was to investigate novel biomarkers associated with aberrant methylation and further develop a reliable predictive test for prognosis of PDAC patients, contributing to more accurate classification of PDAC patients for personalized clinical management. After obtaining 118 methylation-driven genes, functional enrichment and pathway analyses were performed to explore their molecular mechanisms, which revealed that the pathogenicity of DNA methylation aberrations derived from changes in both the cellular mechanisms and the functional interaction among genes. It can be postulated that these genes give rise to PDAC through expression dysregulation afterward, for the methylation-driven genes in PDAC are closely associated with the regulation of transcription.

The method to construct the prognostic model based on the methylation-driven genes followed a three-step process: univariate Cox analysis, lasso regression and multivariate Cox analysis. As a result, a predictive framework for the prognosis of PDAC was developed based on two methylation-driven genes (FOXI2 and MYEOV). Survival analysis demonstrated significant differences in the clinical outcomes of the high and low risk groups, suggesting that this model could produce reliable results on the prognosis of patients with PDAC. The reliability of the prognostic model was further examined by the ROC curve and the AUC predicting 1-year overall survival rate was 0.722, indicating a robust performance in prediction. The decreasing reliability of the Cox model as time grows may be attributed to the extremely short survival time of PDAC patients, typically ranging from 4 to 6 months following diagnosis. PDAC patients of all stages still only retain a 5-year survival below 5% 31, thus the short-term prognostic evaluation is of more clinical value. The Cox model also showed satisfying performance when applied on the GEO datasets, proving its feasibility on other patient groups. This model has practical use in application of predicting prognosis of PDAC patients for the small number of genes involved can simplifies the detention procedure and reduce the cost of patients.

Stratified survival analysis on major clinical factors and risk score proved our model was an independent prognostic factor for PDAC with more accurate outcome compared with traditional methods like stage, grade and TNM classification. This result suggested that our approach is a promising alternative to estimate possible survival rate of PDAC patients. It is also feasible to combine the risk model with the grade for more comprehensive patient data.

MYEOV (myeloma overexpressed) in the model can be useful as an independent prognostic marker and may become novel therapeutic targets. A number of studies have already shown a strong association between MYEOV and multiple neoplasms including esophageal carcinoma 32, oral squamous cell carcinoma 33, neuroblastoma 34, colon cancer 35 and so forth. Recent investigations have demonstrated that the overexpression of MYEOV is associated with poor prognosis of pancreatic cancer patients. An analysis examines TCGA PAAD cohort with the MethHC database revealed a significant reduction in MYEOV promoter methylation in PDAC than that in non-tumoral tissues, leading to MYEOV overexpression. MYEOV promotes pancreatic cancer progression by enhancing transactivity of SOX9, a tumorigenic gene of pancreatic cancer 36. Bioinformatic evidence from a transcriptional study suggests that MYEOV is upregulated in PDAC and associated with poor clinical outcomes, which can be attributed to the facilitation of glycolysis of tumor cells in PDAC 37. Very good agreement was observed when our results of MYEOV being a methylation-related prognostic marker for PDAC was compared to previous findings. MYEOV, whose hypomethylation and high expression were closely associated with low overall survival of PDAC patients, can act as an oncogene in PDAC and can therefore serve as a biomarker for the prognosis of patients with PDAC.

As for FOXI2, its association to tumors has also been investigated. Recent research has demonstrated that FOXI2 promoter methylation may be associated with an increased risk of OSCC development in patients with OPLs 38. It is also found to be aberrantly methylated in most colorectal cancer tissue relative to non-neoplastic tissue 39. Our study is the first one to associate FOXI2 to PDAC, and those previous observations coincided with our findings that FOXI2 was hypermethylated in PDAC and its combined hypermethylation and low expression were indicative of poor prognosis. Since FOXI2 is hypomethylated in peripheral blood DNA, the aberrant methylation of FOXI2 in blood may be a potential biomarker to diagnose PDAC and assess prognosis of patients.

Multiple methylated sites in genes FOXI2 and MYEOV were discovered to have negative correlations with corresponding gene expression and significant associations with the prognosis of PDAC. It can be speculated that aberrant methylation of these sites may contribute to the proliferation and progression of cancers and exert an influence on the prognosis of patients through the disturbance of regular gene expression. Furthermore, by carefully examining the location of methylation sites in the key genes, we discovered none of the two sites in gene MYEOV were related to CpG island (CGI) while the site in gene FOXI2 were all located in CGI. A review of studies concerning methylation and cancer has shown that the emergence of tumor is accompanied by demethylation within multiple genomic regions and de novo methylation of specific CGI 10. Our result demonstrated the expected phenomenon since FOXI2 was hypermethylated and MYEOV was hypomethylated in PDAC. The bulk of prior works in tumor methylation generally concerned themselves with CGI, but our study showed that the prognostic-related sites were not all confined to CGI. Thus, to gain an in-depth understanding of the methylation profile in PDAC, variations of methylation at non- CGI regions should also be emphasized.

This finding of genes and sites methylation in PDAC has a great potential for future clinical application. On account of the early emergence of gene methylation, these methylation-driven genes can be potential indicators for early diagnosis, with studies concerning their sensitivity and specificity reserved for future work. The reversibility of methylation also makes these genes ideal therapeutic targets, enabling novel drugs aiming at methylation-driven genes with prognostic value to modify PDAC development from the very beginning. Considering that one medicine alone is often limited in terms of validity and some methylation alterations can bring about drug resistance, a combination of chemotherapy and methylation-oriented drugs targeting both of FOXI2 and MYEOV may deliver more satisfying results. Diagnosed patients can be evaluated by the risk model with higher accuracy, and later receive personalized treatment based on their estimated prognosis. The methylation level of FOXI2 has already confirmed to be low in blood, further study on the expression and methylation level of MYEOV in blood is suggested. If the tendency of aberrant expression and methylation in blood coincides with that in tissues, it is promising to develop a less invasive reliable detection and evaluation method.

## Conclusion

In this study, methylation-driven genes of PDAC were identified as candidates of detection and prognostic biomarkers. A new method predicting prognosis for PDAC patients based on methylation-driven genes was presented with better performance compared to former approaches. In addition, the expression level of MYEOV and combined methylation and expression degrees of gene FOXI2 and MYEOV in the model were potential prognostic and therapeutic markers for PDAC, and several sites in these genes wielded influences over their prognostic values. Our results came from bioinformatics analysis, thus requiring further cohort studies to validate the findings and to elaborate on the potential clinical utility of such findings.

## Supplementary Material

Supplementary figures and tables.

## Figures and Tables

**Table 1:** GO enrichment analysis of methylation-driven genes in PDAC patients

Category	Term	Count	PValue	FDR
BP	GO:0006351~transcription, DNA-templated	26	4.24E-05	0.026062
MF	GO:0003700~transcription factor activity, sequence-specific DNA binding	15	5.39E-04	0.053228
MF	GO:0000978~RNA polymerase II core promoter proximal region sequence-specific DNA binding	9	6.12E-04	0.053228

Count: the number of enriched genes in the corresponding term. Abbreviations: GO, Gene Ontology; PC, pancreatic cancer; BP, biological process; MF, molecular function.

**Table 2:** The result of univariate Cox analysis

id	HR	HR.95L	HR.95H	pvalue
FOXI2	0.873403	0.820121	0.930147	2.50E-05
CHL1	0.792651	0.683378	0.919398	0.002138
SOX17	0.713766	0.557803	0.913337	0.007349
KRT19	1.376743	1.088055	1.742027	0.007749
LIPH	1.395663	1.091058	1.785309	0.007962
SOWAHC	1.619998	1.125863	2.331006	0.009363
MYEOV	1.219225	1.049659	1.416184	0.009479
NFE2L3	1.444336	1.08047	1.93074	0.013043
LINC01475	0.86643	0.772851	0.971341	0.013949
ID4	0.76494	0.615573	0.950551	0.015628
CHAT	0.939725	0.893229	0.988641	0.016341
AC005498.3	0.804927	0.671318	0.965127	0.019116
CHODL	0.83134	0.711792	0.970966	0.019705
NKX2-3	0.770646	0.618933	0.959547	0.019852
ZNF730	0.91924	0.853125	0.990478	0.027023
KCNA3	0.875242	0.77607	0.987087	0.029871
ZNF382	0.752356	0.579333	0.977053	0.032837
ZSCAN18	0.766412	0.599066	0.980505	0.034294
CERS3-AS1	0.947211	0.900456	0.996394	0.035745
LINC01197	0.833914	0.702521	0.989881	0.037873
NCAM2	0.865201	0.7544	0.992276	0.038372
S100A16	1.345843	1.012746	1.788496	0.040632
SHE	0.812286	0.662708	0.995625	0.045264
IRF4	0.881908	0.778773	0.998703	0.047656

Abbreviation: HR, hazard ratio.
