# Supplementary material for: Analysis of Methylation‐driven Genes in Pancreatic Ductal Adenocarcinoma for Predicting Prognosis
Source: J Cancer. 2021 Sep 9;12(21):6507–18. doi: 10.7150/jca.53208 (PMC8489123; doi:10.7150/jca.53208)
Supplement: Supplementary file 1 — Supplementary figures and tables. [file jcav12p6507s1.pdf]

Additional file 1

Table S1 Details of 118 methylation-driven genes in PDAC.

gene	normal	Tumor	logFC	pValue	adjustP	cor	corPavlue
ADCYAP1	0.110307	0.275374	1.319874	3.82E-06	0.001149	-0.34529	2.57E-05
FOXI2	0.168855	0.38685	1.195987	4.67E-06	0.001404	-0.35039	1.91E-05
IRF4	0.132698	0.283764	1.096547	5.47E-06	0.001647	-0.36449	8.22E-06
NEFH	0.248593	0.415911	0.742486	5.69E-06	0.001714	-0.36352	8.72E-06
ST8SIA3	0.288998	0.405929	0.49017	6.41E-06	0.00193	-0.32909	6.36E-05
NKAPL	0.332391	0.466825	0.490001	6.67E-06	0.002007	-0.54672	1.94E-12
ZSCAN1	0.265113	0.417464	0.655043	6.94E-06	0.002088	-0.39717	9.89E-07
CHAT	0.180224	0.310526	0.784924	7.07E-06	0.002129	-0.34292	2.95E-05
LINC01475	0.222772	0.370477	0.733815	7.80E-06	0.002348	-0.37715	3.72E-06
PHOX2B	0.174849	0.323613	0.888161	7.80E-06	0.002348	-0.32512	7.88E-05
SOX17	0.166359	0.392332	1.237775	8.11E-06	0.002442	-0.48571	8.96E-10
GALR1	0.104979	0.280282	1.416784	9.85E-06	0.002966	-0.37102	5.48E-06
LINC01197	0.340932	0.462171	0.438944	9.85E-06	0.002966	-0.49945	2.50E-10
INA	0.123491	0.311719	1.335834	9.85E-06	0.002966	-0.33045	5.91E-05
TRIM58	0.130361	0.332189	1.349496	1.02E-05	0.003083	-0.43466	6.48E-08
ZNF454	0.147859	0.326659	1.14357	1.02E-05	0.003083	-0.68248	8.53E-21
ABCC9	0.303799	0.419388	0.465166	1.06E-05	0.003204	-0.49412	4.12E-10
ZNF728	0.094072	0.27847	1.565687	1.15E-05	0.003461	-0.41422	2.98E-07
ZNF578	0.220518	0.386632	0.810063	1.19E-05	0.003596	-0.4333	7.20E-08
AF186192.1	0.114514	0.323165	1.496742	1.24E-05	0.003737	-0.42373	1.49E-07
KHDRBS2	0.126266	0.275179	1.123899	1.29E-05	0.003882	-0.38917	1.70E-06
HOTAIRM1	0.171713	0.348938	1.022967	1.29E-05	0.003883	-0.3274	6.98E-05
TMEM196	0.25707	0.42403	0.722005	1.34E-05	0.004034	-0.33164	5.54E-05
SNAP91	0.149573	0.332826	1.153914	1.34E-05	0.004034	-0.34575	2.51E-05
PCDH10	0.119474	0.308874	1.370329	1.34E-05	0.004034	-0.45548	1.23E-08
ZNF382	0.079206	0.290233	1.873529	1.39E-05	0.00419	-0.58744	1.54E-14
KCNJ8	0.155569	0.302282	0.958344	1.39E-05	0.004191	-0.4438	3.17E-08
CCNA1	0.13248	0.281839	1.089097	1.45E-05	0.004353	-0.44713	2.43E-08
PABPC5	0.251016	0.513677	1.033079	1.56E-05	0.004697	-0.52187	2.74E-11
CBLN4	0.252957	0.397958	0.653723	1.62E-05	0.004878	-0.50782	1.11E-10
KDR	0.162354	0.268021	0.7232	1.68E-05	0.005066	-0.35838	1.19E-05
C6orf222	0.70619	0.594789	-0.24768	1.68E-05	0.005066	-0.36351	8.73E-06
SLC6A15	0.118447	0.240575	1.02225	1.68E-05	0.005066	-0.39463	1.18E-06
PSMG3	0.355459	0.231416	-0.6192	1.81E-05	0.005462	-0.48925	6.48E-10
ZNF257	0.093693	0.238971	1.350814	1.81E-05	0.005462	-0.53004	1.17E-11
TPTEP1	0.271804	0.393743	0.534691	1.96E-05	0.005889	-0.44536	2.80E-08
MOGAT2	0.468445	0.339376	-0.46499	2.03E-05	0.006113	-0.32587	7.57E-05
ZNF730	0.064134	0.22898	1.836063	2.03E-05	0.006114	-0.40032	7.97E-07
ID4	0.085128	0.207682	1.286674	2.19E-05	0.006588	-0.44997	1.93E-08
ZNF732	0.253709	0.368657	0.539101	2.19E-05	0.006588	-0.33058	5.87E-05
MEOX2	0.080175	0.170882	1.091786	2.19E-05	0.006588	-0.35907	1.14E-05

TAF1D	0.357793	0.232745	-0.62038	2.36E-05	0.007097	-0.46548	5.32E-09
SFRP1	0.276094	0.378856	0.456488	2.36E-05	0.007097	-0.40356	6.36E-07
GRIA4	0.312063	0.448965	0.524766	2.36E-05	0.007097	-0.31733	0.000119
ZNF208	0.230847	0.381897	0.726242	2.45E-05	0.007365	-0.63019	4.36E-17
ZNF492	0.061677	0.220856	1.840292	2.64E-05	0.007932	-0.53956	4.25E-12
ZNF702P	0.162854	0.248974	0.612414	2.73E-05	0.00823	-0.45973	8.65E-09
ZSCAN18	0.308276	0.42627	0.467541	2.73E-05	0.00823	-0.7405	6.20E-26
CNRIP1	0.199078	0.346632	0.800069	2.73E-05	0.00823	-0.52999	1.18E-11
AC116614.1	0.146181	0.336629	1.203403	2.73E-05	0.00823	-0.37562	4.10E-06
SFN	0.732195	0.53116	-0.46308	2.84E-05	0.008539	-0.51857	3.83E-11
PHYHIPL	0.133728	0.252618	0.917653	2.94E-05	0.008859	-0.38673	2.00E-06
NCAM2	0.244869	0.347809	0.506283	3.05E-05	0.009191	-0.35779	1.23E-05
ZNF135	0.500401	0.601375	0.265182	3.29E-05	0.009889	-0.77321	1.75E-29
CELA3B	0.503737	0.702617	0.480068	3.53E-05	0.010638	-0.56735	1.82E-13
MYEOV	0.434621	0.333317	-0.38286	3.53E-05	0.010638	-0.67494	3.26E-20
EDNRB	0.214976	0.355763	0.72674	3.53E-05	0.010638	-0.49652	3.29E-10
NRK	0.162068	0.400422	1.304921	3.67E-05	0.011032	-0.35257	1.68E-05
CERS3-AS1	0.228186	0.39977	0.808964	3.67E-05	0.011032	-0.31884	0.00011
ZSCAN23	0.104072	0.209103	1.006631	3.67E-05	0.011032	-0.48881	6.75E-10
THBS4	0.421441	0.521649	0.307748	3.67E-05	0.011033	-0.47481	2.37E-09
ZNF418	0.197925	0.344972	0.801522	3.67E-05	0.011033	-0.69909	3.86E-22
CHODL	0.261845	0.405974	0.632676	3.94E-05	0.011863	-0.39339	1.28E-06
CHL1	0.321347	0.440873	0.456231	3.94E-05	0.011863	-0.34841	2.15E-05
NKX2-3	0.114032	0.239654	1.071518	3.94E-05	0.011863	-0.37742	3.65E-06
SRMS	0.61421	0.541835	-0.18088	4.24E-05	0.012753	-0.4931	4.54E-10
KRT19	0.415681	0.305573	-0.44396	4.55E-05	0.013705	-0.58597	1.85E-14
IFI27	0.455007	0.349258	-0.38159	4.55E-05	0.013705	-0.40835	4.54E-07
ZIM2-AS1	0.266842	0.392636	0.557206	5.07E-05	0.015263	-0.32133	9.65E-05
NFE2L3	0.402305	0.270415	-0.57312	5.25E-05	0.015817	-0.48683	8.09E-10
MARVELD1	0.428095	0.302209	-0.50239	5.25E-05	0.015817	-0.53749	5.32E-12
CCDC8	0.310462	0.414623	0.417383	5.45E-05	0.016392	-0.50567	1.37E-10
LINC01354	0.342751	0.533835	0.639235	5.45E-05	0.016392	-0.47543	2.25E-09
ELAVL4	0.478964	0.585166	0.288928	5.64E-05	0.016986	-0.48242	1.21E-09
TBX3	0.25361	0.388965	0.617026	5.85E-05	0.0176	-0.39908	8.68E-07
ZNF471	0.109983	0.300771	1.45139	6.06E-05	0.018235	-0.62495	9.39E-17
S100A16	0.714971	0.561317	-0.34907	6.06E-05	0.018235	-0.51623	4.85E-11
ZNF300P1	0.240114	0.351325	0.549085	6.28E-05	0.018892	-0.43612	5.79E-08
CCBE1	0.168513	0.271031	0.685594	6.28E-05	0.018892	-0.33702	4.11E-05
PHYHD1	0.447499	0.552751	0.304745	6.50E-05	0.019571	-0.39465	1.17E-06
SHE	0.350516	0.43114	0.298677	6.50E-05	0.019572	-0.50021	2.32E-10
VMP1	0.443365	0.318129	-0.47888	6.50E-05	0.019572	-0.37329	4.75E-06
LIPH	0.574991	0.450164	-0.35309	7.23E-05	0.021751	-0.49128	5.37E-10
FITM1	0.427179	0.575372	0.429656	7.75E-05	0.023328	-0.55484	7.78E-13
PCDH17	0.286707	0.393614	0.457205	7.75E-05	0.023329	-0.45721	1.07E-08

PTGDR	0.243754	0.369462	0.599996	8.03E-05	0.024158	-0.39912	8.65E-07
KCNA3	0.334569	0.486956	0.541487	8.03E-05	0.024159	-0.52343	2.33E-11
CNTN1	0.247896	0.360233	0.539195	8.31E-05	0.025016	-0.31757	0.000118
LRFN5	0.096654	0.201753	1.061685	8.31E-05	0.025016	-0.53149	1.01E-11
CUZD1	0.642794	0.813569	0.339907	8.91E-05	0.026816	-0.6638	2.19E-19
C19orf33	0.465284	0.327298	-0.5075	8.91E-05	0.026817	-0.58527	2.02E-14
TBX18	0.138616	0.288353	1.056743	9.06E-05	0.027285	-0.42784	1.09E-07
RNF165	0.231047	0.304963	0.400445	9.22E-05	0.027761	-0.41381	3.07E-07
APOL1	0.371404	0.261191	-0.50788	9.22E-05	0.027762	-0.33578	4.40E-05
ADAM23	0.31084	0.388365	0.321242	9.88E-05	0.02975	-0.32544	7.75E-05
IL2RG	0.513989	0.3719	-0.46682	0.000102	0.030792	-0.38911	1.70E-06
ZNF518B	0.316146	0.449898	0.509007	0.000102	0.030793	-0.68365	6.90E-21
SUSD5	0.356779	0.475713	0.415058	0.000106	0.031869	-0.37757	3.62E-06
AC004009.3	0.547072	0.323503	-0.75795	0.000106	0.03187	-0.39621	1.06E-06
OAS2	0.346239	0.221813	-0.64242	0.00011	0.032981	-0.31934	0.000107
GDPD3	0.595459	0.487005	-0.29007	0.00011	0.032981	-0.41796	2.27E-07
TECPR1	0.667657	0.795054	0.251946	0.000113	0.034131	-0.50531	1.42E-10
TMEM234	0.43664	0.324885	-0.42652	0.000121	0.036546	-0.37105	5.47E-06
ENKD1	0.632846	0.85168	0.428457	0.000123	0.037171	-0.31677	0.000123
CDO1	0.26299	0.379172	0.527845	0.000126	0.037812	-0.62954	4.80E-17
DAPP1	0.590563	0.439168	-0.42732	0.00013	0.03912	-0.53645	5.94E-12
PTGFR	0.21432	0.333603	0.638363	0.000132	0.039789	-0.46549	5.32E-09
CCRL2	0.416466	0.273737	-0.60541	0.000134	0.040469	-0.37039	5.70E-06
CRH	0.345818	0.406486	0.233192	0.000134	0.040469	-0.32913	6.35E-05
C11orf53	0.653889	0.533256	-0.29422	0.000134	0.040469	-0.49139	5.32E-10
OLFM4	0.675459	0.585043	-0.20733	0.000139	0.041862	-0.51737	4.32E-11
KLF6	0.279321	0.166351	-0.7477	0.000139	0.041863	-0.43733	5.27E-08
C15orf62	0.542356	0.370375	-0.55025	0.000139	0.041863	-0.36493	8.00E-06
SOWAHC	0.686679	0.536602	-0.35578	0.000139	0.041864	-0.41631	2.56E-07
SLC25A22	0.651651	0.795471	0.287709	0.000159	0.047903	-0.37748	3.64E-06
KLF2	0.45774	0.28793	-0.66881	0.000159	0.047904	-0.48884	6.73E-10
AC005498.3	0.060288	0.214709	1.832431	0.000162	0.048713	-0.51837	3.91E-11
FAM19A4	0.276755	0.37068	0.421566	0.000165	0.049538	-0.33288	5.17E-05

Normal: the average gene methylation in non-tumoral sample. Tumor: the average gene methylation in tumor sample. FC: fold change. Cor: the correlation coefficient between gene methylation and expression.

Additional file 2

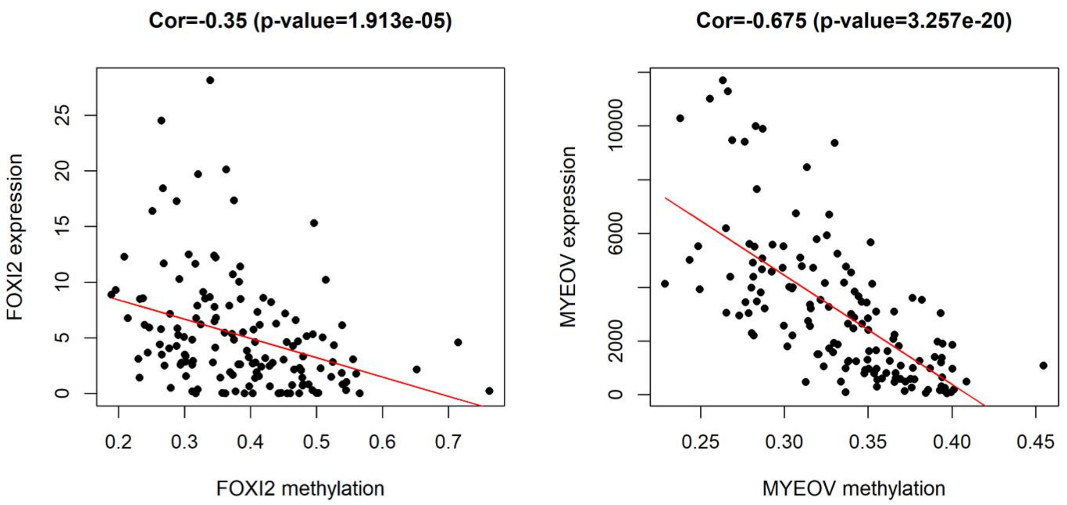

Figure S1 Correlations between the methylation and expression levels of the two key genes.

Additional file 3

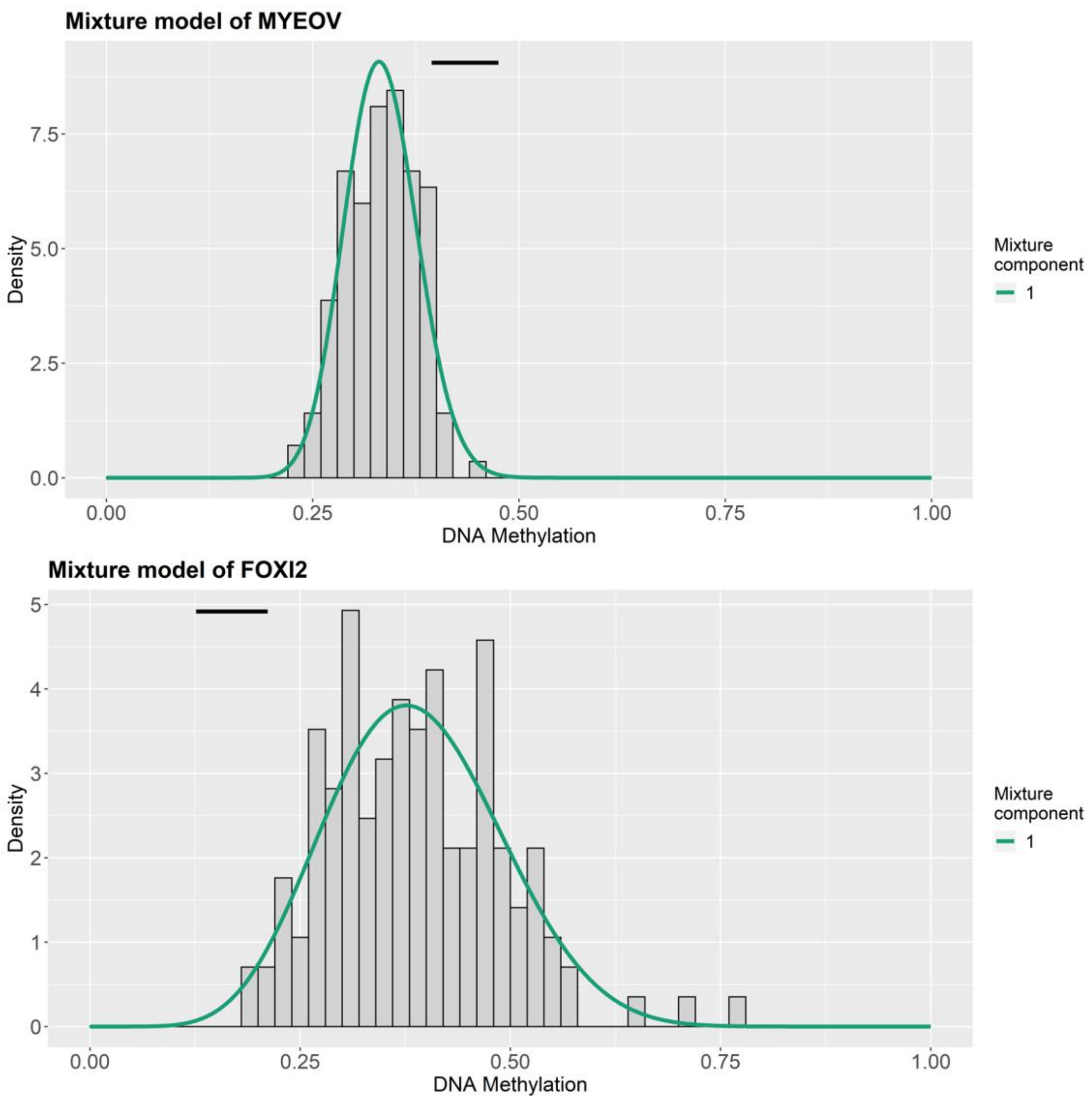

Figure S2 The methylation status of the two key genes in normal and tumor samples.

Additional file 4

Table S2 Detailed clinic data of patients from the TCGA database.

Id	futime	fustat	age	gender	grade	stage	T	M	N
TCGA-2J-AAB6	293	1	75	MALE	G2	Stage IIA	T3	M0	N0
TCGA-2J-AAB8	80	0	71	MALE	G3	Stage IIB	T3	M0	N1
TCGA-2J-AAB9	627	1	70	FEMALE	G1	Stage IIB	T3	M0	N1
TCGA-2J-AABA	607	1	55	MALE	G2	Stage IIB	T3	M0	N1
TCGA-2J-AABE	676	0	73	MALE	G2	Stage IIA	T3	M0	N0
TCGA-2J-AABF	691	1	73	MALE	G3	Stage IIB	T3	M0	N1
TCGA-2J-AABK	484	0	71	MALE	G2	Stage IIB	T3	M0	N1
TCGA-2J-AABO	440	0	43	MALE	G2	Stage IIB	T3	M0	N1
TCGA-2J-AABR	438	0	60	FEMALE	G3	Stage IIA	T3	M0	N0
TCGA-2J-AABT	319	0	72	FEMALE	G2	Stage IIB	T3	M0	N1
TCGA-2J-AABU	277	1	56	MALE	G3	Stage IIB	T3	M0	N1

TCGA-2J-AABV	652	1	74	MALE	G4	Stage IIB	T3	M0	N1
TCGA-2L-AAQA	143	1	76	MALE	G2	Stage IIB	T3	MX	N1
TCGA-2L-AAQE	684	1	56	MALE	G2	Stage IIB	T3	M0	N1
TCGA-2L-AAQI	103	1	66	MALE	G3	Stage IIB	T3	MX	N1
TCGA-2L-AAQJ	394	1	49	FEMALE	G2	Stage III	T4	MX	N0
TCGA-2L-AAQL	292	1	82	MALE	G3	Stage IIB	T3	MX	N1
TCGA-3A-A9I5	1794	0	57	MALE	G1	unknow	unknow	MX	N0
TCGA-3A-A9I7	1323	0	45	MALE	G2	Stage IIB	T3	MX	N1
TCGA-3A-A9I9	634	1	67	MALE	G2	Stage IIA	T3	MX	N0
TCGA-3A-A9IB	224	1	69	FEMALE	G3	Stage IIB	T3	MX	N1
TCGA-3A-A9IC	738	1	61	FEMALE	G2	Stage IIB	T3	MX	N1
TCGA-3A-A9IH	1021	0	66	FEMALE	G2	Stage IA	T1	MX	N0
TCGA-3A-A9IU	458	1	65	MALE	G3	Stage IIB	T3	MX	N1
TCGA-3A-A9IX	1037	0	40	MALE	G2	Stage IA	T1	MX	N0
TCGA-3A-A9IZ	308	1	47	MALE	G2	Stage IIB	T3	MX	N1
TCGA-3A-A9J0	743	0	75	MALE	G2	Stage IIB	T3	MX	N1
TCGA-3E-AAAY	2285	0	67	MALE	G3	Stage IIB	T3	MX	N1
TCGA-3E-AAAZ	2182	1	71	MALE	G2	Stage IIA	T3	MX	N0
TCGA-F2-A7TX	95	1	77	MALE	G3	Stage IIB	T3	M0	N1
TCGA-F2-A8YN	517	0	76	MALE	G2	Stage IIA	T3	M0	N0
TCGA-FB-A4P5	179	1	69	FEMALE	G2	Stage IIB	T3	MX	N1
TCGA-FB-A4P6	363	0	54	MALE	G1	Stage IIB	T3	MX	N1
TCGA-FB-A545	385	0	72	FEMALE	G2	Stage IIB	T3	M0	N1
TCGA-FB-A5VM	449	0	74	MALE	G3	Stage IB	T2	M0	N0
TCGA-FB-A78T	375	1	71	FEMALE	G2	Stage IIB	T3	M0	N1
TCGA-FB-AAPP	485	1	71	MALE	G3	Stage IIB	T3	MX	N1
TCGA-FB-AAPQ	1130	1	65	MALE	G2	Stage IIB	T3	MX	N1
TCGA-FB-AAPS	228	0	62	FEMALE	G2	Stage IIB	T2	MX	N1
TCGA-FB-AAPU	381	1	41	FEMALE	G2	Stage IIA	T3	MX	N0
TCGA-FB-AAPY	1059	1	71	MALE	G2	Stage IIB	T2	MX	N1
TCGA-FB-AAPZ	716	0	54	MALE	G3	Stage IIB	T2	MX	N1
TCGA-FB-AAQ0	473	1	68	MALE	G3	Stage IIA	T3	MX	N0
TCGA-FB-AAQ1	123	1	49	MALE	G2	Stage IIB	T3	MX	N1
TCGA-FB-AAQ2	153	1	81	FEMALE	G3	Stage IIB	T3	MX	N1
TCGA-FB-AAQ3	31	1	65	FEMALE	G2	Stage IIB	T3	MX	N1
TCGA-FB-AAQ6	244	1	85	MALE	G2	Stage IA	T1	MX	N0
TCGA-HV-A5A3	128	1	50	MALE	G2	Stage IIA	T3	MX	NX
TCGA-HV-A5A4	232	0	72	FEMALE	G2	Stage IIA	T3	MX	N0
TCGA-HV-A5A5	289	0	61	FEMALE	G2	Stage IIB	T3	M0	N1
TCGA-HV-A5A6	2036	1	65	FEMALE	G1	Stage IIB	T3	M0	N1b
TCGA-HV-A7OL	252	0	70	MALE	G2	Stage IIA	T3	MX	N0
TCGA-HV-A7OP	978	0	72	MALE	GX	Stage IIB	T3	M0	N1
TCGA-HV-AA8V	920	0	50	MALE	G3	Stage IIB	T3	MX	N1
TCGA-HV-AA8X	532	1	75	FEMALE	G2	Stage IIB	T2	MX	N1

TCGA-HZ-7918	969	0	72	MALE	G3	Stage IIB	T3	M0	N1
TCGA-HZ-7919	593	1	52	FEMALE	G2	Stage IIB	T3	M0	N1
TCGA-HZ-7920	236	1	71	MALE	G2	Stage IB	T2	MX	N0
TCGA-HZ-7922	4	0	61	FEMALE	G1	Stage IIB	T3	MX	N1
TCGA-HZ-7923	314	0	65	MALE	G2	Stage IIA	T3	MX	N0
TCGA-HZ-7924	840	0	60	FEMALE	G2	Stage IIA	T3	MX	N0
TCGA-HZ-7925	614	1	66	MALE	G2	Stage IIB	T3	MX	N1
TCGA-HZ-7926	518	1	57	MALE	G1	Stage IIB	T3	MX	N1
TCGA-HZ-8001	706	0	69	MALE	G2	Stage III	T4	MX	N0
TCGA-HZ-8002	366	1	61	MALE	G2	Stage IIB	T3	MX	N1
TCGA-HZ-8003	596	1	65	FEMALE	G2	Stage IIB	T3	MX	N1
TCGA-HZ-8005	120	1	81	MALE	G3	Stage IIB	T3	MX	N1
TCGA-HZ-8315	299	1	54	FEMALE	G2	Stage IIA	T3	MX	N0
TCGA-HZ-8317	378	1	69	FEMALE	G1	Stage IIB	T3	MX	N1
TCGA-HZ-8636	545	1	58	FEMALE	G3	Stage IV	T3	M1	N0
TCGA-HZ-8637	517	1	76	FEMALE	G3	Stage IIB	T3	MX	N1
TCGA-HZ-A49G	660	0	58	FEMALE	G2	Stage IIB	T2	MX	N1
TCGA-HZ-A49H	491	0	68	FEMALE	G2	Stage IIB	T3	MX	N1
TCGA-HZ-A49I	308	1	77	MALE	G2	Stage IIB	T3	MX	N1
TCGA-HZ-A4BH	194	0	75	MALE	G3	Stage IIB	T3	MX	N1b
TCGA-HZ-A77O	160	1	77	FEMALE	G2	Stage IIB	T2	MX	N1
TCGA-HZ-A8P0	0	0	76	MALE	G1	Stage IIB	T3	MX	N1
TCGA-HZ-A8P1	7	0	81	MALE	G1	Stage IB	T2	MX	N0
TCGA-IB-7644	394	1	65	FEMALE	G2	Stage IV	T3	M1	N1
TCGA-IB-7645	1502	1	44	FEMALE	G1	Stage IIB	T3	M0	N1
TCGA-IB-7646	145	1	60	MALE	G2	Stage IIB	T3	M0	N1
TCGA-IB-7649	467	1	73	FEMALE	G2	Stage IIB	T3	M0	N1
TCGA-IB-7651	603	1	64	FEMALE	G2	Stage IIB	T3	M0	N1
TCGA-IB-7652	724	0	49	FEMALE	G2	Stage IIB	T3	M0	N1
TCGA-IB-7654	476	1	80	MALE	G2	Stage IIB	T2	M0	N1
TCGA-IB-7885	977	0	78	FEMALE	G2	Stage IIB	T3	M0	N1
TCGA-IB-7886	123	1	80	MALE	G3	Stage IIB	T3	M0	N1
TCGA-IB-7887	110	1	62	FEMALE	G2	Stage IIB	T3	M0	N1
TCGA-IB-7888	1332	1	66	FEMALE	G2	Stage IIA	T3	M0	N0
TCGA-IB-7889	481	1	85	FEMALE	G1	Stage IIB	T3	M0	N1
TCGA-IB-7890	598	1	73	MALE	G3	Stage IB	T2	M0	N0
TCGA-IB-7891	648	0	49	FEMALE	G1	Stage IIB	T3	M0	N1
TCGA-IB-7893	117	1	64	MALE	G3	Stage IIA	T3	M0	N0
TCGA-IB-7897	486	1	53	FEMALE	G2	Stage IIB	T3	M0	N1
TCGA-IB-8126	75	0	79	FEMALE	G1	Stage III	T4	M0	N1
TCGA-IB-8127	522	0	59	MALE	G2	Stage IIB	T3	M0	N1
TCGA-IB-A5SO	365	1	71	MALE	G2	Stage IIB	T3	M0	N1
TCGA-IB-A5SP	482	0	77	MALE	G2	Stage IIA	T3	M0	N0
TCGA-IB-A5SQ	219	1	56	FEMALE	G2	Stage IB	T2	M0	N0

TCGA-IB-A5SS	460	1	64	FEMALE	G3	Stage IIB	T3	M0	N1
TCGA-IB-A5ST	635	0	64	FEMALE	G2	Stage IIB	T3	M0	N1
TCGA-IB-A6UF	666	0	63	MALE	G2	Stage IIB	T3	M0	N1
TCGA-IB-A6UG	41	1	65	MALE	G3	Stage IIB	T3	M0	N1
TCGA-IB-A7LX	250	1	57	MALE	G2	Stage IIB	T3	MX	N1
TCGA-IB-A7M4	483	0	81	MALE	G3	Stage IIB	T3	MX	N1
TCGA-IB-AAUM	8	0	76	MALE	G3	Stage IIB	T2	M0	N1
TCGA-IB-AAUN	144	1	74	FEMALE	G2	Stage IB	T2	M0	N0
TCGA-IB-AAUO	239	1	64	FEMALE	G3	Stage IIB	T3	M0	N1
TCGA-IB-AAUP	431	0	68	MALE	G2	Stage IIB	T3	M0	N1
TCGA-IB-AAUQ	183	1	50	MALE	G2	Stage IIB	T3	M0	N1
TCGA-IB-AAUR	338	0	67	MALE	G1	Stage IIB	T3	M0	N1
TCGA-IB-AAUS	225	0	84	FEMALE	G2	Stage IIB	T3	M0	N1
TCGA-IB-AAUU	245	0	35	MALE	G3	Stage IIB	T3	M0	N1
TCGA-IB-AAUV	404	0	49	MALE	G2	Stage IIB	T3	M0	N1
TCGA-IB-AAUW	230	1	63	FEMALE	G3	Stage IIB	T3	M0	N1
TCGA-L1-A7W4	278	1	48	FEMALE	G3	Stage IIB	T3	M0	N1
TCGA-LB-A7SX	393	1	74	FEMALE	G2	Stage IIB	T3	MX	N1
TCGA-LB-A8F3	379	0	64	FEMALE	G1	Stage IIA	T3	MX	N0
TCGA-LB-A9Q5	313	1	63	FEMALE	G3	Stage IIB	T3	MX	N1
TCGA-M8-A5N4	584	0	48	FEMALE	G2	Stage IIA	T3	M0	N0
TCGA-OE-A75W	267	1	75	MALE	G1	Stage IIA	T3	M0	N0
TCGA-PZ-A5RE	470	1	44	FEMALE	G3	Stage IIB	T3	MX	N1
TCGA-Q3-A5QY	416	0	58	MALE	G2	Stage IIB	T3	MX	N1
TCGA-Q3-AA2A	95	0	64	FEMALE	G1	Stage IB	T3	MX	N1
TCGA-RB-AA9M	286	0	43	MALE	G3	Stage IIB	T1	MX	N1
TCGA-RL-AAAS	9	0	60	FEMALE	G2	Stage IB	T2	M0	N0
TCGA-S4-A8RM	646	0	67	MALE	G3	Stage IIB	T3	MX	N1
TCGA-S4-A8RO	525	0	75	FEMALE	G2	Stage IIB	T3	MX	N1
TCGA-S4-A8RP	702	1	77	FEMALE	G3	Stage IIB	T3	MX	N1
TCGA-US-A774	695	1	76	FEMALE	G3	Stage IIB	T3	MX	N1
TCGA-US-A779	511	1	54	FEMALE	G1	Stage IIB	T3	MX	N1b
TCGA-US-A77G	12	1	64	MALE	G2	Stage IIB	T3	MX	N1
TCGA-US-A77J	568	1	81	FEMALE	G2	Stage IIB	T3	MX	N1b
TCGA-XD-AAUG	420	0	66	FEMALE	G2	Stage IV	T3	M1	N1
TCGA-XD-AAUH	395	0	57	FEMALE	G2	Stage IIB	T3	M0	N1
TCGA-XD-AAUI	366	1	50	FEMALE	G2	Stage IIB	T3	MX	N1
TCGA-XD-AAUL	405	0	56	MALE	G2	Stage IIA	T3	MX	N0
TCGA-XN-A8T3	951	0	67	MALE	G2	Stage IB	T2	M0	N0
TCGA-XN-A8T5	720	0	53	FEMALE	G2	Stage IB	T2	M0	N0
TCGA-YB-A89D	350	0	59	MALE	G2	Stage IIB	T3	MX	N1
TCGA-YY-A8LH	2016	0	61	FEMALE	G3	Stage IIB	T3	MX	N1
TCGA-Z5-AAPL	467	0	74	FEMALE	G1	Stage IIA	T3	M0	N0

Additional file 5

Table S5 Clinic data of patients from the GEO database.

geo_accession	futime	fustat	grade
GSM711904	survival_month: 51	cancer_death: 1	
GSM711906	survival_month: 7	cancer_death: 1	
GSM711908	survival_month: 3	cancer_death: 1	
GSM711910	survival_month: 42	cancer_death: 1	
GSM711912	survival_month: na	cancer_death: na	
GSM711914	survival_month: 36	cancer_death: 1	
GSM711916	survival_month: 2	cancer_death: 1	
GSM711918	survival_month: na	cancer_death: na	
GSM711920	survival_month: na	cancer_death: na	
GSM711922	survival_month: 19	cancer_death: 1	
GSM711924	survival_month: 13	cancer_death: 1	
GSM711926	survival_month: 16	cancer_death: 1	
GSM711928	survival_month: 41	cancer_death: 1	
GSM711930	survival_month: 3	cancer_death: 1	
GSM711932	survival_month: 12	cancer_death: 1	
GSM711934	survival_month: 25	cancer_death: 1	
GSM711936	survival_month: 38	cancer_death: 0	
GSM711938	survival_month: 1	cancer_death: 0	
GSM711940	survival_month: 13	cancer_death: 1	
GSM711942	survival_month: 23	cancer_death: 1	
GSM711944	survival_month: 11	cancer_death: 1	
GSM711946	survival_month: 29	cancer_death: 1	
GSM711948	survival_month: 28	cancer_death: 1	
GSM711950	survival_month: 28	cancer_death: 0	
GSM711952	survival_month: 14	cancer_death: 1	
GSM711954	survival_month: 7	cancer_death: 1	
GSM711956	survival_month: 28	cancer_death: 0	
GSM711958	survival_month: 24	cancer_death: 0	
GSM711960	survival_month: 24	cancer_death: 0	
GSM711962	survival_month: 8	cancer_death: 1	
GSM711964	survival_month: 22	cancer_death: 0	
GSM711966	survival_month: 21	cancer_death: 0	
GSM711968	survival_month: 21	cancer_death: 0	
GSM711970	survival_month: 9	cancer_death: 1	
GSM711972	survival_month: 17	cancer_death: 0	
GSM711974	survival_month: 6	cancer_death: 1	
GSM711976	survival_month: 16	cancer_death: 0	
GSM711978	survival_month: 5	cancer_death: 1	
GSM711980	survival_month: 11	cancer_death: 0	
GSM711982	survival_month: 4	cancer_death: 1	
GSM711984	survival_month: 10	cancer_death: 1	

GSM711986	survival_month: 10	cancer_death: 0	
GSM711988	survival_month: 15	cancer_death: 1	
GSM711990	survival_month: 5	cancer_death: 1	
GSM711992	survival_month: 13	cancer_death: 1	
GSM1527105	survival months: 51.1	survival status: 1	grading: G2
GSM1527107	survival months: 6.9	survival status: 1	grading: G3
GSM1527109	survival months: 2.7	survival status: 1	grading: G3
GSM1527111	survival months: 41.6	survival status: 1	grading: G2
GSM1527113	survival months: ?	survival status: ?	grading: G2
GSM1527115	survival months: 35.9	survival status: 1	grading: G2
GSM1527117	survival months: 2.4	survival status: 1	grading: G2
GSM1527119	survival months: ?	survival status: ?	grading: G2
GSM1527121	survival months: ?	survival status: ?	grading: G2
GSM1527123	survival months: 19.5	survival status: 1	grading: G3
GSM1527125	survival months: 12.6	survival status: 1	grading: G3
GSM1527127	survival months: 16	survival status: 1	grading: G2
GSM1527129	survival months: 40.9	survival status: 1	grading: G2
GSM1527131	survival months: 2.8	survival status: 1	grading: G3
GSM1527133	survival months: 11.6	survival status: 1	grading: G3
GSM1527135	survival months: 24.7	survival status: 1	grading: G3
GSM1527137	survival months: 40	survival status: 0	grading: G2
GSM1527139	survival months: 1.2	survival status: 1	grading: G2
GSM1527141	survival months: 13.2	survival status: 1	grading: G2
GSM1527143	survival months: 23.2	survival status: 1	grading: G3
GSM1527145	survival months: 10.8	survival status: 1	grading: G2
GSM1527147	survival months: 29	survival status: 1	grading: G2
GSM1527149	survival months: 27.7	survival status: 1	grading: G2
GSM1527151	survival months: 27.6	survival status: 0	grading: G2
GSM1527153	survival months: 13.8	survival status: 1	grading: G3
GSM1527155	survival months: 6.8	survival status: 1	grading: G3
GSM1527157	survival months: 28.2	survival status: 0	grading: G2
GSM1527159	survival months: 9.8	survival status: 1	grading: G3
GSM1527161	survival months: 23.6	survival status: 0	grading: G3
GSM1527163	survival months: 7.7	survival status: 1	grading: G3
GSM1527165	survival months: 21.8	survival status: 0	grading: G2
GSM1527167	survival months: 21.2	survival status: 0	grading: G4
GSM1527169	survival months: 21.1	survival status: 0	grading: G3
GSM1527171	survival months: 8.9	survival status: 1	grading: G2
GSM1527173	survival months: 17.3	survival status: 0	grading: G3
GSM1527175	survival months: 6.4	survival status: 1	grading: G3
GSM1527177	survival months: 16.4	survival status: 0	grading: G3
GSM1527179	survival months: 4.6	survival status: 1	grading: G2
GSM1527181	survival months: 10.6	survival status: 0	grading: G2
GSM1527183	survival months: 4.2	survival status: 1	grading: G3

GSM1527185	survival months: 10.3	survival status: 1	grading: G2
GSM1527187	survival months: 9.7	survival status: 0	grading: G2
GSM1527189	survival months: 14.9	survival status: 1	grading: G3
GSM1527191	survival months: 4.5	survival status: 1	grading: G3
GSM1527193	survival months: 12.9	survival status: 1	grading: G3
GSM1527196	survival months: 9.5	survival status: 1	grading: G2
GSM1527198	survival months: 6.3	survival status: 1	grading: G3
GSM1527199	survival months: 0.9	survival status: 1	grading: G2
GSM1527200	survival months: 5.9	survival status: 1	grading: G2
GSM1527202	survival months: 9.8	survival status: 1	grading: G3
GSM1527204	survival months: 5.3	survival status: 1	grading: G3
GSM1527205	survival months: 21.5	survival status: 1	grading: G2
GSM1527207	survival months: 14.2	survival status: 1	grading: G3
GSM1527209	survival months: 32	survival status: 1	grading: G3
GSM1527210	survival months: 22.9	survival status: 1	grading: G2
GSM1527212	survival months: 45.9	survival status: 1	grading: G3
GSM1527213	survival months: 21.9	survival status: 1	grading: G2
GSM1527215	survival months: 42	survival status: 0	grading: G2
GSM1527216	survival months: 38.3	survival status: 0	grading: G2
GSM1527218	survival months: 13.7	survival status: 1	grading: G3
GSM1527219	survival months: 10.9	survival status: 1	grading: G2
GSM1527220	survival months: 21.3	survival status: 1	grading: G3
GSM1527223	survival months: 9.3	survival status: 1	grading: G2
GSM1527225	survival months: 19.9	survival status: 1	grading: G3
GSM1527227	survival months: 70.8	survival status: 0	grading: G2
GSM1527228	survival months: 68	survival status: 0	grading: Gx
GSM1527230	survival months: 67.7	survival status: 0	grading: G1
GSM1527232	survival months: 49.7	survival status: 1	grading: G1
GSM1527234	survival months: 3.2	survival status: 1	grading: G2
